# Varietal Tracing of Virgin Olive Oils Based on Plastid DNA Variation Profiling

**DOI:** 10.1371/journal.pone.0070507

**Published:** 2013-08-07

**Authors:** Marga Pérez-Jiménez, Guillaume Besnard, Gabriel Dorado, Pilar Hernandez

**Affiliations:** 1 Instituto de Agricultura Sostenible (IAS-CSIC), Alameda del Obispo s/n, Córdoba, Spain; 2 Laboratoire Evolution & Diversité Biologique (EDB), CNRS-UPS-ENFA, UMR 5174, Bâtiment 4R1b2, Toulouse Cedex 9, France; 3 Dep. Bioquímica y Biología Molecular, Campus Rabanales C6-1-E17, Campus de Excelencia Internacional Agroalimentario (ceiA3), Universidad de Córdoba, Córdoba, Spain; Cankiri Karatekin University, Turkey

## Abstract

Olive oil traceability remains a challenge nowadays. DNA analysis is the preferred approach to an effective varietal identification, without any environmental influence. Specifically, olive organelle genomics is the most promising approach for setting up a suitable set of markers as they would not interfere with the pollinator variety DNA traces. Unfortunately, plastid DNA (cpDNA) variation of the cultivated olive has been reported to be low. This feature could be a limitation for the use of cpDNA polymorphisms in forensic analyses or oil traceability, but rare cpDNA haplotypes may be useful as they can help to efficiently discriminate some varieties. Recently, the sequencing of olive plastid genomes has allowed the generation of novel markers. In this study, the performance of cpDNA markers on olive oil matrices, and their applicability on commercial Protected Designation of Origin (PDO) oils were assessed. By using a combination of nine plastid loci (including multi-state microsatellites and short indels), it is possible to fingerprint six haplotypes (in 17 Spanish olive varieties), which can discriminate high-value commercialized cultivars with PDO. In particular, a rare haplotype was detected in genotypes used to produce a regional high-value commercial oil. We conclude that plastid haplotypes can help oil traceability in commercial PDO oils and set up an experimental methodology suitable for organelle polymorphism detection in the complex olive oil matrices.

## Introduction

The virgin olive oil is obtained by mechanical pressing the fruits of the olive trees (*Olea europaea* L.), and has not undergone any chemical refinement, which is strictly forbidden by law. This product presents excellent organoleptic, nutritional and functional qualities. Its cardiovascular and antioxidant health benefits are widely recognized [1], [2], including a 'qualified health claim’ for coronary heart disease by the Food and Drug Administration (FDA) of the United States of America (2004). It is also a key element of the healthy Mediterranean diet [3].

The olive oil consumption is growing outside the traditional olive tree grove areas (Mediterranean Basin), including America, Asia and Australasia (non-traditional-producer countries such as the United States, Mexico, Brazil, Argentina, Peru, Australia and China; FAO 2012, <http://faostat.fao.org>). Such expansion is mainly due to the recognition of the dietetic properties of olive oil, as source of healthy fatty acids and micronutrients (antioxidants like phenolic compounds, vitamin E, carotenes, etc).

Olive oil is marketed and perceived as a high-quality food product. Additionally, the price of the virgin olive oil is high compared to other edible oils, being therefore considered as a high-value product, which makes it prone to adulteration [4]. Despite some previous publications about this topic (see [5] for a review) the olive oil traceability remains a challenge. This includes both the identification of oils from other species [6], [7], as well as oils from different olive varieties. The European Commission introduced two types of certification labels in 1992, in order to protect the authenticity of the Extra Virgin Olive Oil (EVOO). Such labels refer to food products specific to a particular region or town, conveying a particular quality or characteristic of the specified area. Namely, they are the Protected Designation of Origin (PDO) and the Protected Geographical Indication (PGI) (EEC Regulation No. 2082/92). A further EEC Regulation (No. 510/2006) specifies the criteria for labeling, production and commercial distribution of the olive oil.

Accurate analytical approaches have been then developed to help the identification of genuine olive oil constituents and possible adulterants, the cultivar and the geographical origin. Thus, chromatographic and spectroscopic/spectrometric techniques have been used to analyze the content of metabolites such as triacylglycerols, free fatty acids, phenols (like hydroxytyrosol), sterols, alkanes, waxes and aliphatic alcohols [8]–[10]. Nevertheless, the content of metabolites can be affected by the environmental conditions during the plant growth, which might cause ambiguous or erroneous results [11]. Therefore, the chemical analyses are not enough for themselves to verify the olive oil authenticity or its varietal identification.

On the other hand, different genomic DNA molecular markers have been developed for olive cultivar identification during the last decade. Among them, only nuclear markers such as genomic microsatellites (gSSR; [12], [13]), Sequence-Characterized Amplified Regions (SCAR; [14], [15]), and Amplified Fragment Length Polymorphisms (AFLP; [16]), already used for the characterization of olive tree cultivars, have been proposed for the varietal traceability of the olive oil [17]. Success in the varietal authentication of the olive oil has been reported using AFLP ([18], [19], [20]), SCAR ([21]), gSSR ([22], [23], [24], [25], [26]), and nuclear single nucleotide polymorphisms (SNP; [27]). DNA-based molecular markers are indeed the best choice for traceability purposes, since they are not dependent on the environmental and processing conditions, unlike other chemical analyses based on metabolites detection (see [28] for a review).

The nuclear microsatellites (gSSR) have been the molecular markers of choice for authenticity purposes. So much, that they are the only molecular markers accepted in the courts worldwide. This is due to several facts, including their codominant nature, high polymorphism conferring to them a high-discriminating power, wide distribution across the genome, automated detection and simple interpretation. Yet, the genomic microsatellite markers should be used with caution in the case of monovarietal olive oil traceability, due to the presence of paternal alleles from the seed [29].

The plastid genome has some advantages in relation to the nuclear genome for traceability purposes [30]: i) it is maternally inherited; ii) thousands of copies are present per cell, which is an extremely significant advantage for forensic analyses; iii) it is circular instead of linear, and therefore resistant to exonucleases; iv) organelles have a double membrane that makes chloroplast DNA more resistance to degradation, which is also a significant advantage for forensics; and v) it has a lower mutation rate than nuclear genomic sequences, and its stability may be an advantage for traceability analyses, despite of a low polymorphism level. Plastid markers have been already used to detect adulteration of olive oil, but only to analyze mixtures of oils from different species [31]. However, the varietal identification of the olive oils through molecular markers based in variations of the plastid genome has not been reported so far. This is due to the fact that the cpDNA polymorphism between olive tree varieties was not enough characterized, and therefore not sufficient to develop useful molecular markers for olive oil traceability. To solve this problem, we have previously sequenced eight plastid genomes of *Olea* [30]. This has allowed to develop a set of molecular markers to characterize the cpDNA of cultivated and wild Mediterranean olive trees. As expected, the discriminating power of the cpDNA variation was particularly low for the cultivated olive trees, being higher for the oleasters (wild olives).

Based on the above developments, the objective of the present work is to evaluate the efficiency of a subset of nine cpDNA regions [that include microsatellites, small insertions/deletions (indels) or a combination of both] for the varietal identification of both leaf DNA and the corresponding oil DNA, further assessing their applicability and contribution to the traceability and authenticity of the olive tree varieties and their monovarietal oils. Thus, we selected 15 major Mediterranean olive cultivars and two locally exploited trees (referred as “acebuchinas”) from the southern of Spain (Andalusia). The cultivars are already included in the PDO oil catalogue or in the process of obtaining such recognition <http://ec.europa.eu/agriculture/quality/door/list.html> (Table 1).

**Table 1:** Olive varieties analyzed.

Cultivar	Country	Source	Type	PDO region^b^	Use	Commercial monovarietal oil
Arbequina	Spain	IFAPA	Monovarietal^a^	Campo de Montiel, **Comunitat Valenciana**, Aceite de laRioja, Aceite de Mallorca, **Aceite de Navarra**, Aceitedel Bajo Aragón, Antequera, Les Garrigues, **Lucena**,Sierra de Cádiz, Siurana, Aceite de Terra Alta,Sierra de Cádiz	oil, table	Yes
Blanqueta	Spain	Intercoop	Commercial	**Comunitat Valenciana**, Aceite de la Rioja	oil	Yes
Farga Canetera	Spain	Intercoop	Commercial	Aceite del Baix Ebre-Montsià, Aceite de Terra Alta,**Comunitat Valenciana,** Baix Ebre-Montsià	oil	Yes
Farga Milenaria	Spain	Intercoop	Commercial	Aceite del Baix Ebre-Montsià, Aceite de Terra Alta,**Comunitat Valenciana**	oil	Yes
Frantoio	Italy	IFAPA	Monovarietal^a^	Umbria, Sabina, Colline Pontine, Colline di Romagna,Collina di Brindisi, Irpinia-Colline dell’Ufita, Collina diTeatine, Collina di Salernitane, Monti Iblei, Garda	oil, table	Yes
Galega Vulgar	Portugal	IFAPA	Monovarietal^a^	Azeite do Alentejo Interior	oil	Yes
Gordal Sevillana	Spain	IFAPA	Monovarietal^a^	NA	table	no
Hojiblanca	Spain	IFAPA	Monovarietal^a^	Antequera, Baena, Estepa, Lucena, Poniente de Granada,Priego de Córdoba, Sierra de Cádiz	oil, table	Yes
Lechín de Sevilla	Spain	IFAPA	Monovarietal^a^	Antequera, Baena, Lucena, Montoro-Adamuz,Sierra de Cádiz	oil	Yes
Manzanilla de Sevilla	Spain	IFAPA	Monovarietal^a^	Campo de Montiel, Aceite de la Rioja, Sierra de Cádiz	oil, table	Yes
Picual	Spain	IFAPA	Monovarietal^a^	Campo de Calatrava, Campo de Montiel, Aceite de laRioja, Aceite de Mallorca, Aceite Monterrubio, Antequera,Baena, Estepa, Lucena, Montes de Granada, Montoro-Adamuz,Poniente de Granada, Priego de Córdoba, Sierra de Cádiz,Sierra de Cazorla, Sierra de Segura,Sierra Mágina	oil, table	Yes
Picholine Languedoc	France	IFAPA	Monovarietal^a^	Huile d'Olive de Nîmes, Huile d'Olive de Haute-Provence	oil, table	Yes
Toffahi	Egypt	IFAPA	Monovarietal^a^	NA	table	no
Villalonga	Spain	IFAPA	Monovarietal^a^	**Comunitat Valenciana**	oil	Yes
Zaity	Syria	IFAPA	Monovarietal^a^	NA	oil	NA
Acebuchina 2	Spain	El Callejón	Monovarietal^a^	NA	oil	Yes
Acebuchina 5	Spain	El Callejón	Monovarietal^a^	NA	oil	Yes

List of olive oil varieties used showing information about country of origin, olive or oil suppliers, type of olive oil used for DNA extraction, protected denomination of origin to which oils belong, the use of olives for table or for making oil and if monovarietal oil is commercialized.

a Abencor small-scale production;

b NA: not available.

Boldface: Commercial olive oils in the process of obtaining PDO recognition.

## Materials and Methods

### Plant Materials and Commercial Monovarietal Olive Oil

This study was performed with 15 cultivated olive trees and two “acebuchinas” (Table 1). The leaves and drupes from selected olive trees were collected during the 2010/2011 harvest from single plants. Most plant materials were provided by the ‘Olive World Germplasm Bank’ (OWGB) at the “Centro Alameda del Obispo” of the “Instituto Andaluz de Investigación y Formación Agraria, Pesquera, Alimentaria y de la Producción Ecológica” (IFAPA; Córdoba, Spain). The leaves and olive oil samples from ‘Blanqueta’, ‘Farga Milenaria’ and ‘Farga Canetera’ denominations were supplied by the Mill Cooperative Intercoop (Almazora, Castellón, Spain). The ‘Picholine Languedoc’ and ‘Farga’ leaves were received from the Olive Tree Germplasm Bank at the Institute for Olive Tree and Subtropical Plants of Chania, National Agricultural Research Foundation (NAGREF; Agrokipio, Chania, Greece). The ‘acebuchina’ leaves and drupes were supplied by the olive-growing cooperative ‘El Callejón’ (Cádiz, Spain).

### Olive Oil Production

Monovarietal olive oils were produced using 3 to 5 kg of olive drupes from certified trees. The physical extraction procedure used (Abencor System) is certified to be equivalent to the one used in production olive mills, and was carried out using an Olive Oil Efficiency Analyzer (Hammer Mill, ThermoMixer and Centrifuge) from MC2 Ingeniería y Sistemas (Seville, Spain, [32]). Briefly, the olive fruits were washed and the leaves removed, within 24 to 48 h after sampling. The olives were crushed with a hammer mill and slowly mixed for 30 to 60 min at 25°C. Natural talc and warm water were added to increase the oil yield during the mixing. Then, in order to separate the solid from the liquid phases, the obtained paste was centrifuged at 1,000 *g* for 1 min, followed by decantation of the oil. Finally, the oil was transferred into dark glass bottles and stored at room temperature in the dark until the DNA extraction.

### DNA Extraction from Leaves and Olive Oils

The genomic DNA from leaf tissues was extracted according to the CetylTrimethylAmmonium Bromide (CTAB) method [33] as optimized by [34]. The DNA extraction from the ‘Blanqueta’, ‘Farga Milenaria’ and ‘Farga Canetera’ monovarietal commercial olive oils, as well as the ones generated by the Olive Oil Efficiency Analyzer were carried out using a modified CTAB method [18]. All the oil DNA extractions were carried out in duplicate, in order to obtain enough DNA for the necessary amplifications and PCR replicates. All the olive oil DNA extractions used in this work were carried out from fresh olive oils within a four-month period. Previously, molecular marker set-up has been carried out with three-year-old commercial olive oil from the 'Picual' variety with similar results.

### Molecular Analyses

Nine Polymerase Chain Reaction (PCR) primer pairs developed for the olive plastid genomic profiling [30] were used (Table 2). We did not use an 18-bp tail of M13 on the forward primer as reported by [30] because we amplified each locus separately. Briefly, the PCR mixtures contained: i) the DNA isolated from either leaves or olive oils; ii) the reaction buffer made of 200 mM Tris-HCl (pH 8.3 at 25°C), 200 mM KCl, 50 mM (NH_4_)_2_SO_4_ and 3 mM MgCl_2_; iii) 25 mM of each dNTP; iv) 0.6 U of Hot Start *Taq* DNA polymerase from Fermentas (part of Thermo Fisher Scientific; Glen Burnie, MD, USA); and v) the PCR primers, including 50 pM of the forward primer fluorescently labeled with either 6-FAM, HEX or NED fluorochrome (Table 2), and 50 pM of the reverse primer. For locus 10, the reverse primer was HEX-labeled instead of the forward primer.

**Table 2:** Plastid markers, variable motifs and PCR primers used.

Locus name	Motif	Forward primer (5′–>3′)^a^	Reverse primer (5′–>3′)	Amplicon size range (bp)
1	polyT_10–13_	AAAGGAGCAATAACGCCCTC	GGATAAGACCCGATCTTAGTG	99–101
10	indel 1 bp+(ATTAGATA)_1–2_	AAGGRGTCTTTCTTTCTCTATTC	TAGGCTCGTTCGAGCCCTTC	81–89
19	polyC_10–11_+T_9–11_+A_12–15_	TTATTTCAGTTCAGAGTTCCTCC	CCAAATTGATGTTCCAATATCTTC	89–91
51	polyT_11–18_	GGTGAACTAAAATTATGGGTGC	TAGATTGTGTCTCACGCATATAC	117–125
27	polyA_8–11_	CTCGGTTATGAGACACATTACAAT	CAAGAAGTTTGCAAGAAGTTTGAC	107–108
38	polyT_10–11_	AACAAGATTGTTTAGATCTGATGG	TCGAAATAGATATCTGTGTTATGC	104–105
46	polyA_10–12_	AATAGCATGGCACTTCGAATTC	ATCTCATACTACTCTCTCGATAC	108–109
57	polyA_13–15_+ indel 1 bp	CAATATGAAATGGAATTCGCTCC	ATTGTAACAAATAGGGAGATGCG	221–224
11	indel 10 bp+polyA_11–14_	AGATAAAGGAAGGGCTCGAACG	CAGGCCATCAGAATAAGAAGGG	103–114

Data from Besnard et al. [30].

a Forward primers were FAM-HEX or NED-labeled, except for locus 10, in which the reverse primer was labeled with HEX.

The reaction mixtures (15 µl) were incubated in a MyCycler thermocycler from Bio-Rad (Hercules, CA, USA) at 95°C for 2 min (denaturation), followed by 36 cycles (95°C for 30 s for denaturation, 57°C for 30 s for annealing, and 72°C for 1 min for extension). The reaction was finally extended at 72°C for 20 min and stored at –20°C until use. The PCR products generated (amplicons) were visualized under blue light using a DR195M “Dark Reader” transilluminator from Clare Chemical Research (Dolores, CO, USA), after 2% (w/v) agarose gel electrophoresis and staining with GelGreen from Biotium (Hayward, CA, USA). The amplicons were further segregated by capillary electrophoresis using an ABI Prism 3130xl Genetic Analyzer from Life Technologies (Carlsbad, CA, USA), using the GeneScan 3.7 software from the same manufacturer.

For some loci, PCR reactions from oil DNA were repeated up to four times to ensure robust allele determination (see below). An allele size was considered as robust when the peak signal was of a high quality (as for PCR reactions from leaf DNA) and thus allowed a non-ambiguous allele determination.

## Results

The nine cpDNA loci used amplified DNA isolated from both olive leaves and oils and were able to discriminate six allele combinations or haplotypes (Table 3). Most cultivars showed haplotypes E1-1 (‘Arbequina’, ‘Frantoio’, ‘Hojiblanca’, ‘Manzanilla de Sevilla’ and ‘Picual’) and E1-2 (‘Galega Vulgar’, ‘Gordal Sevillana’, ‘Toffahi’ and ‘Zaity’). The remaining cultivars harbored haplotypes E1-3 (‘Blanqueta’ and ‘Villalonga’), E2-1 (‘Picholine Languedoc’), E2-3 (‘Lechín de Sevilla’, ‘acebuchina-2’ and ‘acebuchina-5’) and E3-1 (‘Farga Milenaria’ and ‘Farga Canetera’).

**Table 3:** Plastid DNA haplotype for each olive variety and Locus-allele combinations for each olive variety.

Haplotype [30]	Allele combination	Variety
E1-1	1–101, 19–90, 51–125, 10–89, 27–107, 38–104	Arbequina, Frantoio, Hojiblanca, Manzanilla, Picual
	46–109, 57–224,11–103	
E1-2	1–101,19–91, 51–124, 10–89, 27–107, 38–104	Galega Vulgar, Gordal Sevillana, Toffahi, Zaity
	46–109, 57–224, 11–103	
E1-3	1–101, 19–90, 51–124, 10–89, 27–107, 38–104	Blanqueta, Villalonga
	46–109, 57–224, 11–103	
E2-1	1–100, 19–89, 51–117, 10–81, 27–107, 38–105	Picholine Languedoc
	46–108, 57–221, 11–114	
E2-3	1–100, 19–89, 51–117, 10–82, 27–107, 38–105	Lechín de Sevilla, Acebuchina 2, Acebuchina 5
	46–108, 57–221, 11–114	
E3-1	1–99, 19–89, 51–125, 10–89, 27–108, 38–105	Farga Milenaria, Farga Canetera
	46–109, 57–222, 11–112	

**Figure 1:**
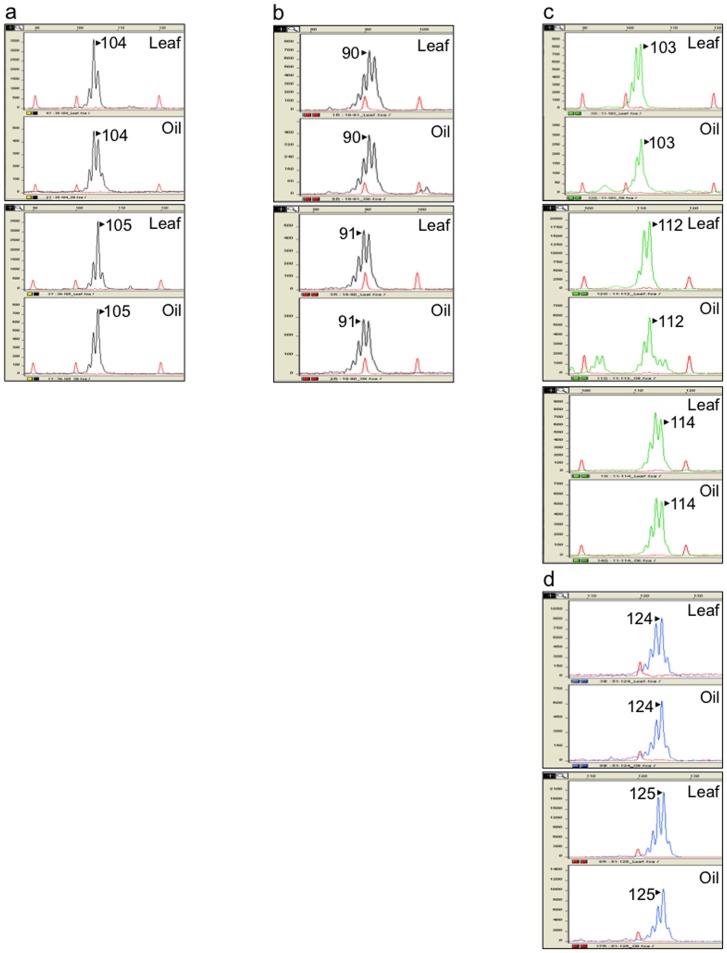
Profiling of olive plastid DNA markers. Examples of chromatograms showing congruent DNA amplification from leaves (up) and oils (down). The allele peaks are marked with the corresponding allele size (bases). **a**) locus 38; **b**) Locus 19; **c**) Locus 11 and **d**) Locus 51.

**Figure 2:**
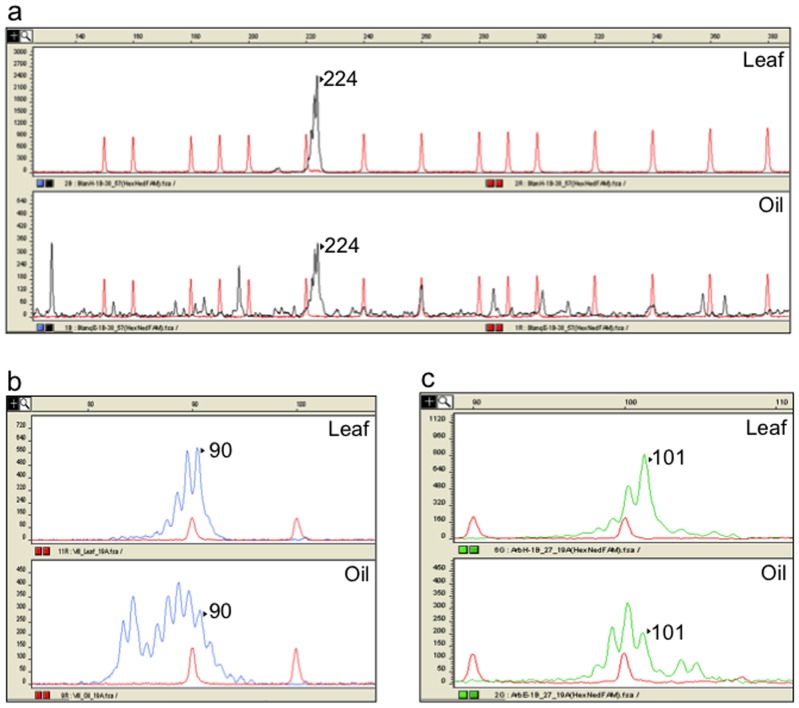
Discrepancies found between oil and leaf amplification patterns. Examples of chromatograms showing discrepancies in DNA amplification from leaves (up) and oil (down) for **a**) locus 57 on variety ‘Blanqueta’; b) locus 19 on variety ‘Villalonga’; **b**) locus 1 on variety ‘Arbequina’. The expected allele peaks (as defined on leaf DNA) are marked with the corresponding allele size (bases).

Several patterns of PCR amplification were found. Six loci (10, 11, 27, 38, 46 and 51) generated clear amplicons, with a high quality of peak signal, corresponding to a single DNA fragment of the expected size for DNA isolated from both olive leaf and oil (Figure 1a–d). Occasionally, the other three cpDNA loci (1, 19 and 57) showed somewhat discordant results between leaf and oil samples. Locus 57 generated unspecific amplifications, shown on the chromatograms as a background with several low-intensity peaks for DNA from oil, which did not interfere with allele scoring (Figure 2a). On the other hand, locus 1 produced specific amplifications for leaf DNA, but the oil samples' chromatograms revealed an additional unspecific peak close to the true allele. This additional peak resulted in a poor resolution and intensity of the true allele in the profile of recovered oil DNA (Figure 2c). Finally, locus 19 showed the most complicated scored pattern on amplifications from olive oil DNA. This included the presence of unspecific peaks in the chromatograms, showing similar areas and heights than the true enclosed allele, which hampered the assignation of the correct molecular weight (Figure 2b). Nonetheless, it should be emphasized that such results were not obtained on all oil DNA amplifications. Actually, locus 19 includes three successive microsatellite motifs (polyC/polyT/polyA) and Besnard et al. [30] recommended not using it in a PCR multiplex due to difficulties of amplification.

Whereas locus 57 only needed to be repeated for DNA of ‘Blanqueta’ oil, loci 1 and 19 required up to three and four replications, respectively, of several oil sample amplifications to ensure a robust allele determination (Table S1). For both loci, the number of amplifications required was variable among oil samples. Only for the ‘Picholine Languedoc’ sample, one amplification reaction was sufficient to confidently score alleles of oil and leaves for both loci, followed by ‘Zaity’, with one amplification required for locus 1 and two amplifications in the case of locus 19. We have not observed, however, any correlative trend among the olive oil variety and the number of sample repetitions required. Despite of results obtained on these three loci, they are not all indispensable to discriminate the six haplotypes on oil DNA. Only the use of locus 19 is unavoidable as is the only available marker capable to differentiate the E1-2 and E1-3 haplotypes [30], and its 'inconsistency' problem can be overcome with a 4-replication of the amplification of the oil DNA samples.

## Discussion

### Implications of Nuclear vs. Plastid Molecular Markers for Olive Oil Traceability

Genomic SSRs have been largely used for traceability and authenticity of several foodstuffs [4]. Indeed, they have been considered as a powerful tool to characterize and identify the olive oil varieties and the best option for olive oil traceability. Yet, they have some drawbacks that should be taken into account. The virgin olive oils are the juice of the whole olive fruit (usually drupes with their stones) after being crushed in the milling process. Therefore, the paternal alleles from the seed are mixed with the maternal ones from the mesocarp tissue, being therefore present in the DNA recovered from the olive oil. In any case, it is clear that the paternal genome is present in the DNA isolated from olive oil, albeit at low concentrations, and although it may not be detectable in some cases, it cannot be excluded. Therefore, the nature of the nuclear molecular markers could lead to the misinterpretation of the results. On the contrary, since the chloroplasts are maternally inherited in olive [35], there is no risk of paternal genome contamination. In this study, we detected six haplotypes when we analyzed DNA extracted from leaves. When analyzing the DNA amplifications on oil extracts, we have not observed extra alleles in the scorable profiles, and thereby we conclude that using plastid markers prevents such a problem.

Unexpected alleles in nuclear marker profiles from monovarietal olive oils have been previously reported [29]. In agreement with this, the presence of extra-alleles has also been mentioned and debated in several works. For example, extra-alleles as in stoned ‘Coratina’ olive oil samples were found with the SSR marker GAPU89 [26] and presence of additional alleles was found in monovarietal oils ‘Carolea’, ‘Frantoio’, ‘Leccino’, ‘Nocellara’ and ‘Coratina’ using SSR GAPU59 [36]. Similar mismatches have been described in the ‘Chemcheli Gafsa’ and ‘Arbequina’ oils using SSR UDO09 [37]. Other reports have not found allelic differences between olive oils obtained from stoned and destoned drupes [38]. Yet, such result is not surprising, and could be explained if the approach was not sensitive enough to detect it. Nevertheless, the presence of paternal DNA is not always an insurmountable obstacle, as the potential pollinators of PDO areas could be traced [28], but only when their number remains limited. Special care should be taken in areas with presence of oleaster populations.

### Amplification Specificity

In most cases, the amplification profile was the same for DNA from leaves and from olive oil of each particular variety (some examples are shown on figures 1a–d). Loci 10 and 11, along with microsatellites 27, 51, 38 and 46 have shown a good performance, with an easily-scored pattern, allowing discriminating five out of the six haplotypes on DNA isolated from olive oils, in agreement with the results previously described for olive trees [30]. Therefore, these molecular markers can be used for both genotyping cultivated and wild olive trees, as well as for discrimination analyses of olive oils (Table 3).

Sometimes, loci 1, 19 and 57 showed unclear amplification profiles when amplified from oil samples (Figs. 2a–c). Notwithstanding, the non expected peaks were easily identifiable and non-repetitive across the amplifications. On the other hand, for a given primer pair, the amplificability was not alike for the olive oil DNA of different varieties (data not shown). This could be attributed to chemical differences between the olive oils, including inhibitors that could interfere with the PCR. Indeed, we have found that different unwanted compounds as polyphenols and polysaccharides may co-precipitate in the process of olive oil extraction, depending on the olive tree variety (data not shown).

These facts, in combination with the presence of degraded DNA and the primer design limitations may explain some inconsistencies between leaf and oil DNA amplifications (i.e., due to difficulties to confidently score allele size on some loci for oil olive PCR), as other authors have also found [25]. This is expected, due to the potential difficulty to isolate and amplify DNA from olive oil, which is mostly an hydrophobic substance with tiny amounts of water droplets from the olive fruit juice extraction process (where the DNA resides). In our study, as described in the 'Materials and Methods' section, the inconsistencies were primer-dependent and were present in three of the nine selected plastid markers (loci 1, 19 and 57). For these three loci, the amplification reactions had to be replicated in order to obtain a confidently scorable pattern of alleles on olive oil DNA. It is important to note that the scorable alleles always matched with the alleles scored on the corresponding olive plant leaf. From these three markers, only locus 19 is necessary for the differentiation of the six haplotypes.

Previous studies with genomic SSR molecular markers have also found unspecific amplifications as well as missing alleles [22], [37], [38]. Other authors have found a correspondence in the genotyping profiles between DNA isolated from olive leaves and oils on up to 50% for a total of 222 comparisons [26]. There is a general agreement that mis-amplification and drop-out alleles are due to a low DNA concentration coupled with an excessive degradation of such DNA in the olive oil. Interestingly, we have not observed any allelic drop-out. This may be due to the fact that the cpDNA is more easily recoverable from the olive oil than the nuclear DNA for the reasons outlined above [30], which further supports its advantage for forensic studies. In addition, only one cpDNA allele is expected to be amplified excluding the possibility of drop-out alleles due to competitive amplification of alleles with different size as on nuclear loci.

Additionally, since the olive oil contains both scarce amounts of highly degraded DNA, due to the hydrophilic nature of the DNA in an hydrophobic matrix, the olive oil extraction procedure and the time of storage after milling [39], and the possible presence of inhibitors and other substances that may hinder the PCR performance, the amplification of short amplicons (e.g., about 200 bp or shorter) is highly recommended, as previously demonstrated in forensic DNA studies. Indeed, the advantage of using primers generating shorter SSR products with more robust amplifications has been previously reported when comparing standard and shorter amplicons for nuclear SSR loci DCA14 and EMO30 on both olive leaf and oil [26]. Therefore, PCR primers were designed to amplify short DNA segments (87 to 224 bp; [30]), and they generated robust PCR amplifications in most cases (Table 2 and Fig. 1).

### The Utility of Rare Haplotypes

The selected loci for this work correspond to non-coding regions, which are more likely to show variations due to neutral random mutation events. However, the polymorphism detected was low in the eight olive tree plastid genomes sequenced [30], being not enough to assign each cultivar to a different haplotype (Table 3). For instance, Besnard et al. [30] have shown that ‘Frantoio’ and ‘Manzanilla de Sevilla’ have exactly the same chloroplast genome sequence probably due to a shared ancestral maternal origin in the Near East [40]. Here, the 15 analyzed monovarietal olive oils could be classified into six haplotypes, with ‘Farga’ (‘Milenaria’ and ‘Canetera’), ‘Picholine Languedoc’ and ‘Lechín de Sevilla’ being associated to unique ones. Actually, a dataset of cpDNA haplotypes is already available for 534 olive cultivated genotypes from all the Mediterranean countries [40]. While 80% of cultivars show haplotype E1.1 (which is thus not really useful to discriminate varieties), it was shown that haplotypes E1.3 (‘Blanqueta’ and ‘Villalonga’), E2.1 (‘Picholine Languedoc’), E2.3 (‘Lechín de Sevilla’) or E3.1 (‘Farga’) are rare in cultivated olive (with frequency inferior to 5%). Therefore, these rare haplotypes may be used for traceability of such olive oil varieties. In our study, we have also included two local accessions referred as 'acebuchinas' in this study (Table 1), since they are currently used for the production of commercial olive oil in southern Spain, due to their relevant dietetic properties, including organoleptic, and healthy ones, like their antioxidative potential. Besides, being olive trees with small fruits, their yield is very low, and thus especially prone to fraudulent mixing with other oils. They also represent interesting candidates to assess the possibility of finding new alleles, since more cpDNA variation is expected in local varieties, particularly in potential olive last glacial maximum refugia such as Andalusia [40]. Indeed, this approach allowed to determine that the local ‘acebuchinas’ showed haplotype E2-3. On the 534 Mediterranean cultivars [40], this haplotype was detected only once (in ‘Lechín de Sevilla’). It thus displays a high discriminating power, and the use of our cpDNA markers can easily allow detection of frauds in this case.

### Concluding Remarks

**Figure 3:**
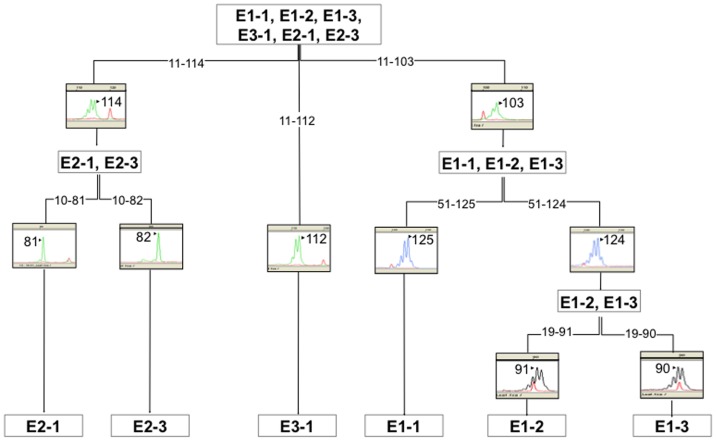
Flowchart outlining one of possible approaches to identify the six haplotypes described in the present study. The flowchart indicates the different steps to be taken for the discrimination of the six analyzed haplotypes.

In summary, the main goal of this work has been to ascertain the utility of cpDNA molecular markers for the development of a methodology to assess the authenticity of olive oil, allowing the identification of the PDO and PGI labels. Based on our results, it was possible to establish that four loci are enough to properly classify cultivars into the six haplotypes described here. One of the possible combinations using loci 11, 10, 51 and 19, is shown in Figure 3. To our knowledge, this is the first report of the development of molecular markers based on cpDNA polymorphisms for the traceability of the olive oils. The described methodology can be used for the varietal traceability of four commercial oils, three of them belonging to recognized PDO. Our results can be helpful to complement other molecular analyses based on nuclear polymorphisms, contributing towards the development of a reference dataset of molecular markers for the Mediterranean olive trees, including both cultivated and wild varieties.

## Supporting Information

Table S1 **Replications required for achieving suitable patterns for oil DNA for loci 1, 19 and 57 per olive variety.**
(PDF)
