# Supplementary material for: Varietal Tracing of Virgin Olive Oils Based on Plastid DNA Variation Profiling
Source: PLoS One. 2013 Aug 7;8(8):e70507. doi: 10.1371/journal.pone.0070507 (PMC3737381; doi:10.1371/journal.pone.0070507)
Supplement: Table S1 — Replications required for achieving suitable patterns for oil DNA for loci 1, 19 and 57 per olive variety. (PDF) [file pone.0070507.s001.pdf]

Table S1. Replications required for achieving suitable patterns for oil DNA for loci 1, 19 and 57 per olive variety.

Variety	Locus		
	1	19	57
Arbequina	3	2	1
Blanqueta	2	4	2
Farga Canetera	2	3	1
Farga Milenaria	2	2	1
Frantoio	2	2	1
Galega Vulgar	2	2	1
Gordal Sevillana	3	3	1
Hojiblanca	3	3	1
Lechín de Sevilla	3	2	1
Manzanilla de Sevilla	2	2	1
Picual	3	1	1
Picholine Languedoc	1	1	1
Toffahi	1	4	1
Villalonga	2	3	1
Zaity	1	2	1
Acebuchina 2	2	4	1
Acebuchina 5	2	4	1
